# Supplementary material for: z-STED Imaging and Spectroscopy to Investigate Nanoscale Membrane Structure and Dynamics
Source: Biophys J. 2020 Apr 16;118(10):2448–57. doi: 10.1016/j.bpj.2020.04.006 (PMC7231928; doi:10.1016/j.bpj.2020.04.006)
Supplement: Document S1. Supporting Materials and Methods and Figs. S1–S5 [file mmc1.pdf]

Biophysical Journal, Volume *118*

Supplemental Information

z-STED Imaging and Spectroscopy to Investigate Nanoscale Membrane Structure and Dynamics

Aurélien Barbotin, Iztok Urbančič, Silvia Galiani, Christian Eggeling, Martin Booth, and Erdinc Sezgin

Supplementary Information

Membrane motion observed with imaging and FCS

In cells, membrane motion limited the maximum observation time in FCS measurements. Indeed, the membranes of living cells move with time. Because of the extremely small axial size of the Gaussian observation volume (approximately 100 nm FWHM), even small displacements of the membrane induce significant artefacts in FCS curves. To evaluate this, we recorded a series of 6 movies of the top and bottom membranes of Ptk2 cells (50 frames acquired with a framerate of 3.2 s, at a STED power of 50 mW). In each movie, we manually selected three points in each membrane, the position of which was measured by Gaussian fitting (Figure S1, A-C). Plotting the mean displacement with time (Figure S1D) and comparing it with the displacement measured in a SLB revealed that cellular membranes move significantly more than SLBs. We quantified this displacement with linear fits, the slope of which we used as an estimate of membrane motion (0.7 and 0.6 nm/s median shift for top and bottom respectively, while only 0.01 nm/s for SLBs). Non-zero intercepts were caused by the limited localisation precision induced by noise. The changes in intensity resulting from varying membrane position within the focus precluded long z-STED-FCS measurements in cells (Figure S1E), while no such effect was visible in SLBs (Figure S1F).

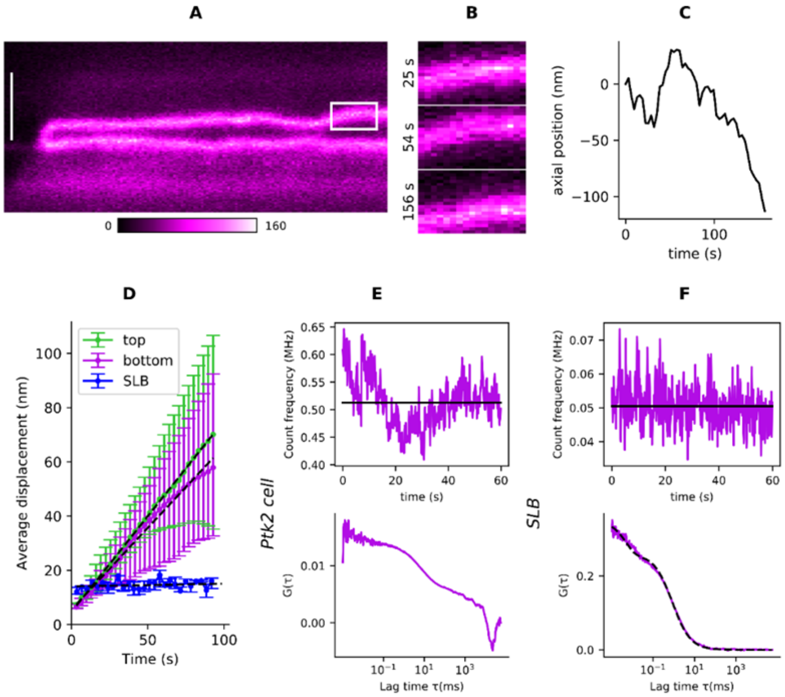

Figure S1. Characteristic drift observed with imaging (A-D) and FCS (E, F). A) First frame of a movie used to measure membrane motion, acquired with STED in PtK2 cells. B) Zoom on the area highlighted in A), at different time points as indicated on the left. C) Time-dependent axial position of the area highlighted in A). D) Average membrane displacement measured as a function of time, in the top and bottom membranes of PtK2 cells and in SLBs as indicated in the legend (median +/- interquartile range, cells: n = 18 points in 6 cells, SLB: n = 3 points in 1 SLB). Dotted black lines: linear fits. E-F) z-STED-FCS curves acquired for 60 s in E) the top membrane of a Ptk2 cell and F) in an SLB.

Representative FCS curves

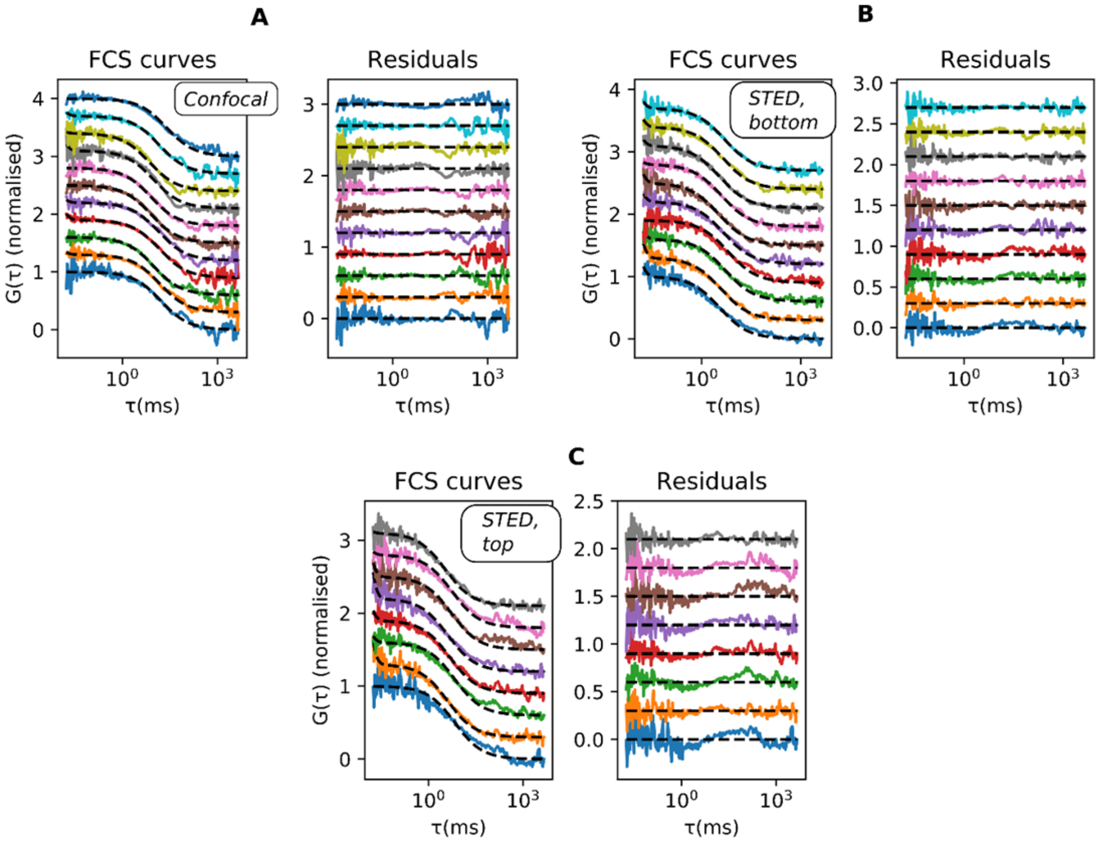

Figure S2. FCS curves (left) and corresponding residuals (right) acquired in cells, in A) confocal mode, B) STED in the bottom membrane and C) STED in the top membrane.

Image deconvolution

Shallow side lobes due to undepleted fluorescence signal were particularly visible when imaging membranes with z-STED. A solution to remove them is to use image deconvolution. First, the point spread function (PSF) of the microscope was estimated. From images of SLBs, we estimated the axial intensity profile of the z-STED PSF (see Figure 2C in main text). The lateral intensity profile was assumed to be Gaussian, with a lateral size estimated from FCS recordings in SLBs estimated to be equal to 160 nm (see Methods section). The PSF was then reconstructed as follows:

$$PSF(x,z) = I(z)\exp(-4\log(2)x^2/\omega^2) \quad (1)$$

Where *I(z)* is the axial intensity profile and ω the lateral FWHM of the PSF. Using this PSF (Figure S1A), images were deconvolved employing the Richardson-Lucy algorithm (20 iterations) in Python using the library scikit-image[1]. Deconvolution improved the sharpness of the images and efficiently suppressed the side lobe contributions (Figure S3).

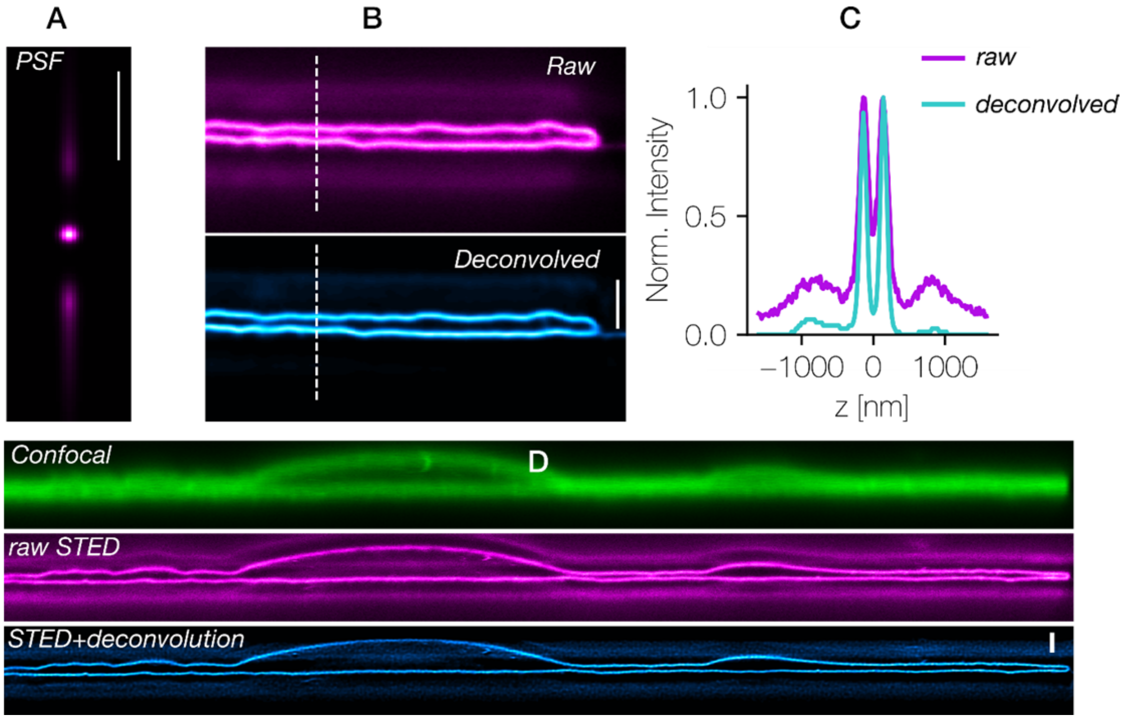

Figure S3. Suppressing contribution from side lobes due to undepleted fluorescence signal using image deconvolution. A) PSF of the z-STED microscope, estimated from imaging and FCS of SLBs. B, C) Raw (magenta, top B) and deconvolved (blue, bottom B) of z-STED images of two axially close-by membranes along with intensity profiles (C) along the line marked in B. D) Deconvolution of a z-STED image of a whole Ptk2 cell: comparison of confocal (top, green), raw STED (middle, magenta) and deconvolved z-STED (bottom, cyan). Scale bars 1 µm.

z-STED imaging of fluorescent lipids in different cell types

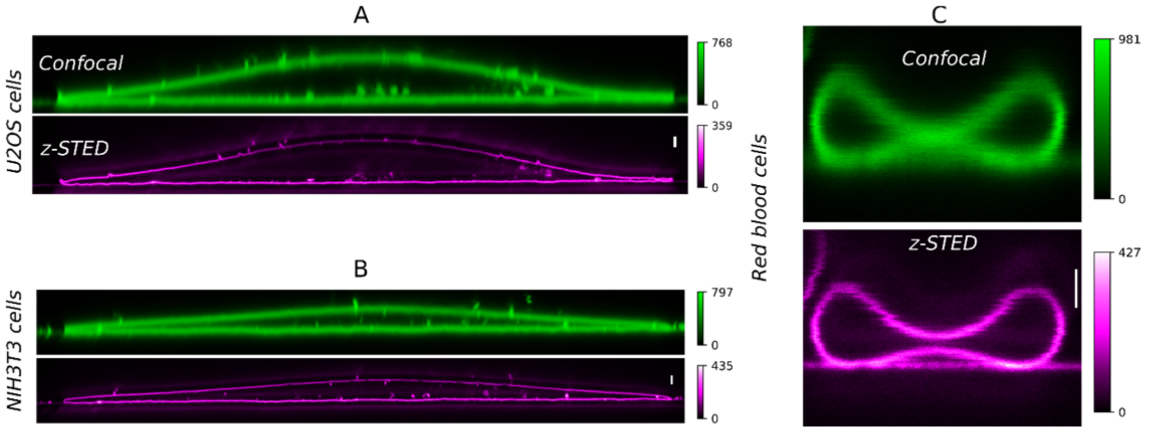

Figure S4. Imaging different cell types with z-STED. *xz* images of live A) U2OS, B) NIH3T3, and C) red blood cells, labelled with Abberior Star Red-PEG-Cholesterol. Scale bars 1 µm.

***xy* improvement of GP imaging**

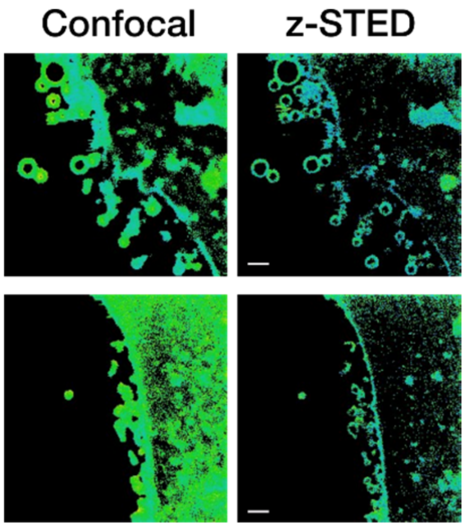

Figure S5. *xy* confocal and STED GP images of cells labelled with NR12S probe, showing how increase in contrast in axial direction helps visualizing and characterizing the small vesicles that cannot be resolved with confocal. Scale bars are 1 µm.

Theoretical analysis of FCS experiments in two close-by membranes

We compared the results obtained when doing FCS in the top and bottom membranes with z-STED as well as on both at the same time with confocal FCS. It is possible to analytically predict the outcomes of such measurements, if they satisfy three assumptions:

1. The intensity fluctuations in the top and bottom membranes are statistically independent.
2. The top and bottom membranes are close enough so that the confocal cross section is identical at both positions.
3. The label concentration and diffusion speed of the top and bottom membranes are strictly identical.

Following these assumptions, the time-dependent intensity recorded in confocal mode by the detector can be written as:

$$I(t) = I_t(t) + I_b(t) \tag{2}$$

Where *I(t)* is the time-dependent intensity recorded by the detector, $I_t(t)$ is the intensity emitted at the top membrane and $I_b(t)$ the intensity emitted at the bottom membrane.

The autocorrelation function of the time trace fluctuations writes as:

$$G(\tau) = \frac{\langle \delta I(t) \delta I(t+\tau) \rangle}{\langle I(t) \rangle^2} \tag{3}$$

where $\langle . \rangle$ denotes the time-averaging operator, and $\delta I(t) = I(t) - \langle I(t) \rangle$ represents the temporal intensity fluctuations. Replacing equation 2 in equation 3 and expanding yields:

$$G(\tau) = \frac{\langle \delta I_t(t) \delta I_t(t+\tau) \rangle + \langle \delta I_b(t) \delta I_b(t+\tau) \rangle + \langle \delta I_t(t) \delta I_b(t+\tau) \rangle + \langle \delta I_b(t) \delta I_t(t+\tau) \rangle}{\langle I_t(t) \rangle^2 + \langle I_b(t) \rangle^2 + 2\langle I_b(t) \rangle \langle I_t(t) \rangle} \tag{4}$$

This expression can be greatly simplified using our above assumption. Independence of the fluctuations in the top and bottom membranes (assumption 1) involves that $\langle \delta I_t(t) \delta I_b(t+\tau) \rangle = \langle \delta I_b(t) \delta I_t(t+\tau) \rangle = 0$. Assumptions 2 and 3 state that the average intensities and intensities fluctuations are identical in top and bottom membranes and we can conclude that $\langle I_t(t)^2 \rangle = \langle I_b(t)^2 \rangle$ and $\langle \delta I_t(t) \delta I_t(t+\tau) \rangle = \langle \delta I_b(t) \delta I_b(t+\tau) \rangle$. As such, equation 4 can be simplified as:

$$G(\tau) = \frac{2\langle \delta I_t(t) \delta I_t(t+\tau) \rangle}{4\langle I_t(t) \rangle^2} = \frac{1}{2} G_t(\tau) \tag{5}$$

Where $G_t(\tau)$ is the autocorrelation function obtained if only the top (or bottom) membrane was present. Equation (5) thus means that FCS measurements performed on two axially close-by membranes leaves the transit times (and consequently apparent diffusion coefficient) unchanged, but divides the amplitude by a factor two. This means that the apparent number of fluorescent molecules is twice higher when measuring two membranes at once than when

measuring membranes separately. This matches our experimental results (Figure 4, H and I in main text).

References

1. Stéfan van der Walt, Johannes L. Schönberger, Juan Nunez-Iglesias, François Boulogne, Joshua D. Warner, Neil Yager, Emmanuelle Gouillart, Tony Yu and the scikit-image contributors. scikit-image: Image processing in Python. PeerJ 2:e453 (2014)
