# Supplementary material for: z-STED Imaging and Spectroscopy to Investigate Nanoscale Membrane Structure and Dynamics
Source: Biophys J. 2020 Apr 16;118(10):2448–57. doi: 10.1016/j.bpj.2020.04.006 (PMC7231928; doi:10.1016/j.bpj.2020.04.006)
Supplement: Document S2. Article plus Supporting Material [file mmc2.pdf]

Biophysical Journal
Article

z-STED Imaging and Spectroscopy to Investigate Nanoscale Membrane Structure and Dynamics

Aurélien Barbotin,[1] Iztok Urbančič,[2,3] Silvia Galiani,[2,4] Christian Eggeling,[2,4,5,6] Martin Booth,[1] and Erdinc Sezgin[2,7,*]

[1]Department of Engineering Science and [2]MRC Human Immunology Unit, MRC Weatherall Institute of Molecular Medicine, University of Oxford, Oxford, United Kingdom; [3]Jožef Stefan Institute, Ljubljana, Slovenia; [4]Wolfson Imaging Centre Oxford, MRC Weatherall Institute of Molecular Medicine, University of Oxford, Oxford, United Kingdom; [5]Institute of Applied Optics and Biophysics, Friedrich Schiller University Jena, Jena, Germany; [6]Leibniz Institute of Photonic Technology e.V., Jena, Germany; and [7]Science for Life Laboratory, Department of Women's and Children's Health, Karolinska Institutet, Solna, Sweden

ABSTRACT Super-resolution stimulated emission depletion (STED) microcopy provides optical resolution beyond the diffraction limit. The resolution can be increased laterally (*xy*) or axially (*z*). Two-dimensional STED has been extensively used to elucidate the nanoscale membrane structure and dynamics via imaging or combined with spectroscopy techniques such as fluorescence correlation spectroscopy (FCS) and spectral imaging. On the contrary, *z*-STED has not been used in this context. Here, we show that a combination of *z*-STED with FCS or spectral imaging enables us to see previously unobservable aspects of cellular membranes. We show that thanks to an axial resolution of ~100 nm, *z*-STED can be used to distinguish axially close-by membranes, early endocytic vesicles, or tubular membrane structures. Combination of *z*-STED with FCS and spectral imaging showed diffusion dynamics and lipid organization in these structures, respectively.

SIGNIFICANCE We report a simple optical system to obtain an ~100-nm axial resolution, which allows us to obtain extremely sharp images of nearby membranes. We combine this technology with spectral methods such as spectral imaging or fluorescence correlation spectroscopy to characterize the biophysical properties of such membranes. Owing to its versatility and excellent axial resolution (which can be combined with lateral super-resolution), this technology will find a wide range of applications in cell and membrane biology.

INTRODUCTION

Cellular membranes are hubs for cellular signaling (1). They are heterogeneous structures accommodating clusters, domains, and nanoassemblies (2,3), and this heterogeneity is crucial for cellular signaling (4). Therefore, there has been extensive effort to resolve the mystery of nanoscale structure and dynamics of cellular membranes. Super-resolution imaging technologies have been extremely useful for shedding light on the nanoscale architecture and the supramolecular organization of the cells (5–11).

Super-resolution can be used to increase not only the lateral but also the axial resolution of microscopes. Single-molecule switching microscopes achieved axial resolutions in the order of magnitude of tens of nanometers (12–14) or even down to 1 nm with recent technologies (15), yet at a relatively slow speed, limiting live-cell applications. Similar localization precision could be achieved using metal-induced energy transfer (16–19); however, this method allows measuring localizations only close to a coverslip. Stimulated emission depletion (STED) microscopy does not suffer from these shortcomings despite having a lower axial resolution (20). Besides, STED can be combined with spectroscopic tools to probe the physical and chemical properties of membranes with nanoscale resolution (21–24). Fluorescence correlation spectroscopy (FCS) is such a spectroscopic tool used to measure molecular mobility in membranes (25,26), which is an important parameter to understand the molecular dynamics in cells (25–30). The combination of STED with FCS (STED-FCS) has been used extensively to address the nanoscale membrane structure (31–37). Recently, STED has also

Submitted January 10, 2020, and accepted for publication April 6, 2020.
*Correspondence: erdinc.sezgin@ki.se
Editor: Gerhard Schuetz.
https://doi.org/10.1016/j.bpj.2020.04.006
© 2020 Biophysical Society.
This is an open access article under the CC BY license (http://creativecommons.org/licenses/by/4.0/).

been combined with spectral imaging and polarity-sensitive probes to quantitatively study the nanoscale physiochemical properties of the membrane (21).

In the context of membrane research, so far, only a two-dimensional (2D) depletion scheme increasing the lateral (xy) resolution has been used in STED-enhanced spectroscopic measurements. This prevented applications in systems having close-by features along the optical axis (z), such as the cellular top and bottom membranes or plasma membrane and internal membranes. A different depletion scheme can be used to increase mostly the axial resolution, relying on a "bottle-shaped" beam for depletion (we will call it z-STED hereafter) (38). The z-STED depletion pattern has been used both alone and together with 2D STED (three-dimensional (3D) STED) for imaging (20,39–44) and STED-FCS (45–47) in solution or cytoplasm. However, the exacerbated sensitivity to aberrations (48,49) and difficulty of operation of the z-STED depletion pattern have prevented its widespread use. Recently, spatial light modulators (SLMs) have been used in STED microscopy systems to mitigate these challenges through aberration correction (43,50–52) and bespoke calibration protocols (53).

In this study, we used SLM-based z-STED with an axial resolution of $\sim$100 nm to study the nanoscale structure and dynamics of the cell membranes. We show that axially close-by membranes or early endocytic vesicles can be distinguished and studied using z-STED imaging. Moreover, the axial resolution of z-STED allowed extremely sharp optical sectioning, which we used to image membrane tubular structures unresolvable by confocal or 2D-STED imaging. Finally, we used a combination of z-STED and FCS to measure diffusion dynamics and combined them with spectral imaging to assess the lipid organization in close-by membranes.

MATERIALS AND METHODS

Bead sample

Microscope slides of 40-nm far-red fluorescent beads were purchased from Abberior Instruments (Göttingen, Germany).

Preparation of supported lipid bilayers

Supported lipid bilayers (SLBs) were prepared with a spin coater (54). The coverslips were cleaned with piranha solution (3:1 sulfuric acid and hydrogen peroxide) beforehand. 1 mg/mL 1-palmitoyl-2-oleoyl-sn-glycero-3-phosphocholine in chloroform/methanol (with 0.01 mol% of Abberior STAR RED-labeled phosphatidylethanolamine (Abberior Instruments)) was spin coated on to a clean coverslip at 3200 rpm for 30 s. The lipid film was rehydrated with SLB buffer (10 mM HEPES and 150 mM NaCl (pH 7.4)).

Cells and maintenance and staining

All cells were maintained at 37°C and 5% CO_2. PtK2 cells were grown in Dulbecco's Modified Eagle's Medium (Sigma-Aldrich, St. Louis, MO) supplemented with 15% fetal bovine serum (Sigma-Aldrich) and 1% L-glutamine (Sigma-Aldrich). NIH-3T3 and U2OS were grown in Dulbecco's Modified Eagle's Medium (Sigma-Aldrich) supplemented with 10% fetal bovine serum (Sigma-Aldrich) and 1% L-glutamine (Sigma-Aldrich). Red blood cells were obtained from mouse blood. Cells were labeled with the fluorescent lipid Abberior Star Red-PEG-Cholesterol in phenol-red-free L15 medium (Sigma-Aldrich) at a concentration of 0.2 μg/mL for 3–5 min at room temperature. After washing twice with L15, measurements were performed also in L15 medium at room temperature. Each slide was imaged not longer than 30 min.

Optical setup

We used a custom STED microscope implemented around a commercial RESOLFT microscope from Abberior Instruments described in detail in (55), to which we added an SLM (Hamamatsu LCOS X10468-02 (Hamamatsu Photonics, Hamamatsu City, Japan)) in the depletion path, as described in (52). Depletion was ensured by a laser (pulse stretched by a 40-cm glass rod and a 100-m single-mode fiber; Spectra-Physics Mai Tai, Santa Clara, CA) pulsing at a frequency of 80 MHz at a wavelength of 755 nm. STED imaging and FCS were performed using a 640-nm pulsed diode laser (PicoQuant, Berlin, Germany) pulsing at a frequency of 80 MHz. Polarity-sensitive dyes for spectral imaging were excited with a 485-nm pulsed diode laser (PicoQuant). The microscope was equipped with an oil immersion objective lens (UPLSAPO, 100×/1.4 oil; Olympus, Tokyo, Japan). STED laser power was set to 110 mW and measured in the back focal plane of the objective.

Alignment of the system

Residual system aberrations in the depletion path of the microscope were removed using the SLM. The depletion beam was imaged by scanning the focus through a sample of scattering gold beads, and the amount of system aberrations present was determined using the sensorless method, using the image SD as image quality metric. Coalignment between the SLM pupil and the objective back aperture is critical (56), and we ensured this at the beginning of each experiment by inspecting the depletion pattern using scattering gold beads. The detailed SLM-STED calibration protocol we used can be found in (53).

Image acquisition and processing

Images were acquired with a pixel size of typically 40 nm in the lateral direction and 20 nm in the axial direction and were later resized using Python or ImageJ. Pixel dwell times varied between 40 and 160 μs.

FCS measurements and fitting

The STED microscope was equipped with a hardware correlator from correlator.com (Flex02-08D) operated by Flex software. Abberior STAR RED dyes (Abberior Instruments) were excited with a 640-nm laser at an excitation power of 2–5 μW. The excitation beam was focused on membranes by varying its axial position to maximize the signal. In cells, the acquisition time of FCS curves was set to 5 s to limit the impact of membrane motion through the observation focus (see Fig. S1). The resulting signal levels were sufficient to obtain good FCS curves (Fig. S2). This was not a problem in SLBs, for which acquisition times were set to 10 s. FCS curves were fitted using a custom Python script. The observation area (defined as the area in which fluorophores emit light contributing to the correlating signal) was assumed to be Gaussian. A 2D diffusion model including a triplet state was used to fit the data (57).

$$G(\tau) = \frac{1}{N}\left(1 + \frac{T}{1-T}e^{-\tau/\tau_T}\right)\frac{1}{1+\tau/\tau_{xy}}, \qquad (1)$$

where N is the average number of molecules in the observation area, T is the average triplet amplitude, τ_T is the triplet correlation time set to 5 μs, and τ_{xy} is the average lateral transit time in the observation area. Diffusion coefficients (D) were determined from FCS transit times (23):

$$D = \frac{\omega^2}{8\ln(2)\tau}, \tag{2}$$

where ω is the full width at half maximum (FWHM) of the Gaussian observation area and τ is the average molecular transit time in the observation area determined with FCS. The FWHM of the confocal observation area was determined from images of immobilized fluorescent beads and set to 240 nm. To reliably compare FCS measurements obtained with confocal and STED, we estimated the increase in lateral STED resolution with FCS using SLBs. Assuming free diffusion, the same diffusion coefficient is expected in both STED (D_s) and confocal (D_c):

$$D_c = D_s. \tag{3}$$

Given Eqs. 2 and 3, the Gaussian lateral FWHM w_s of the STED focus can be estimated as follows:

$$\omega_s = \omega_c\sqrt{\frac{\tau_s}{\tau_c}}. \tag{4}$$

The average number of molecules in the observation area N determined from fitting of individual FCS curves was divided by the measured size of the observation area to fairly compare confocal and STED recordings. Finally, the number of molecules in the observation area was normalized with the confocal value:

$$N_{norm} = \frac{N/\omega^2}{N_c/\omega_c^2}. \tag{5}$$

In SLBs, N_c was set to the average value of all confocal recordings. In cells, the average number of molecules was normalized separately in each cell to account for variations in label concentrations between different cells.

Spectral imaging

We stained the cells with 0.5 μM NR12S (a dye that partitions to the outer leaflet of the plasma membrane and reports on the membrane lipid packing (58)) in L15 media for 5 min at room temperature. Then, the cells were washed twice. Imaging was performed in L15 media at room temperature. Each slide was imaged no longer than 30 min. We have collected the green channel signal with a 510- to 590-nm filter (I_G) and the red channel filter with a 650- to 730-nm filter (I_R). Images were analyzed with the FiJi general polarization (GP) plugin using Eq. 6 (59):

$$GP = \frac{I_G - I_R}{I_G + I_R}. \tag{6}$$

RESULTS AND DISCUSSION

Axial resolution improvement with *z*-STED

The most common implementation of STED makes use of a ring-shaped focus (“doughnut”; Fig. 1 *A*) created by modulating the phase of the depletion laser with a vortex phase mask. STED imaging with this mask (2D STED) increases the lateral resolution (Fig. 1 *B*) but leaves axial resolution unchanged (Fig. 1 *D*). In our microscope, the phase mask was created by an SLM, and as such, it could be swapped to any other phase mask to change the STED confinement mode without changing the optical layout. Using a top-hat phase mask, a bottle-shaped depletion pattern (Fig. 1 *C*) could be created that mainly increases the axial resolution (Fig. 1 *D*) but also slightly increases the lateral resolution (Fig. 1 *B*). The *z*-STED depletion pattern is more challenging to use than its 2D counterpart in part because of its exacerbated sensitivity to spherical aberrations, which we mitigated here by imaging only at shallow depths (0–4 μm). Certain STED microscopes use a combination of the 2D and *z*-STED depletion patterns to increase the resolution along all dimensions (called 3D STED). This requires further experimental complexity because the centers of the two depletion patterns must be coaligned with a nanometric precision. Besides, in the presence of coma aberrations, the centers of the 2D and *z*-STED depletion patterns move in opposite directions, which significantly deteriorates signal levels and resolution (60). In this work, mainly axial resolution improvement was sought, and therefore, *z*-STED was preferred over 3D STED.

Resolving adjacent membranes with *z*-STED

We first estimated the performance of our *z*-STED microscope on lipid membranes using a SLB, which is a single lipid bilayer deposited on a glass surface. This membrane is ~5–8-nm thick, which is well below the expected axial resolution of the *z*-STED microscope and is therefore an excellent sample to estimate the axial resolution. In confocal imaging, the FWHM of the axial Gaussian intensity profile was 854 ± 27 nm, whereas *z*-STED reduced it to 108 ± 5 nm (Fig. 2, *A* and *B*). Undepleted side lobes created a shadow image at ~800 nm above and below the membrane; however, it was at a much lower intensity that can easily be eliminated by image deconvolution (see Fig. S3). In the presence of two close-by membranes, such as in PtK2 cells, the increased axial resolution of *z*-STED allowed us to easily resolve cellular top and bottom membranes as close as 150 nm (Fig. 2, *C–H*). Similar observations could be made in different cell types such as NIH-3T3 cells, U2OS cells, or red blood cells (Fig. S4). Finally, we used the capability of *z*-STED to resolve axially close-by membranes to image layered (grown on top of each other) cells (Fig. 2, *F–H*), revealing once again details in images that were inaccessible to confocal images only. These structures could be crucial for cellular communication and organization.

Resolving submicron structures with *z*-STED

Submicron structures are common in biology and can have various topologies. For example, submicron tubular

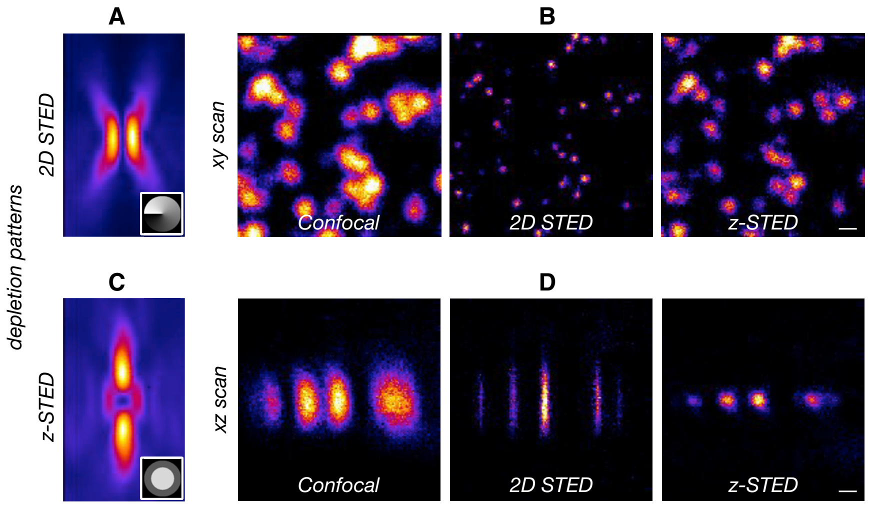

FIGURE 1 2D and z-STED confinement modes. (*A* and *C*) Shown are the depletion beams of (*A*) 2D STED and (*C*) z-STED visualized by scanning a sample of scattering gold beads through the depletion focus. The insets shown are phase masks used to create the depletion patterns (*gray* to *black color scale* for 0 to 2π phase delay). (*B* and *D*) Shown are the *xy* (*B*) and *xz* (*D*) images of immobilized fluorescent beads, imaged with confocal (*left*), 2D STED (*middle*), and z-STED (*right*). Scale bars represent 250 nm. To see this figure in color, go online.

structures are crucial for cellular communications (61). They cannot usually be studied with conventional fluorescence microscopes, the resolution of which is insufficient to differentiate, for instance, between full and hollow membrane tubes. Such multilayer membrane patches are common in SLBs, even though it is not clear how these patches can exist despite the hydrophobic repulsion of the lipid acyl chains at the edges of these patches. One possible scenario is the formation of nanotubes at the edges of these patches. We verified this hypothesis using the increased axial resolution provided by z-STED. Imaging the multilayer SLB patches with z-STED revealed tubular structures at the edges (Fig. 3 *A*), which could not be resolved in confocal images. We further visualized the networks of smaller patches in SLBs, which all turned out to be submicron nanotubes (Fig. 3 *B*). The axial (*xz*) image cross sections revealed the tubular nanostructures with great precision. Additionally, this depletion scheme also significantly improved image contrast, which was extremely helpful for *xy* images of these structures. When we acquired lateral (*xy*) images and compared the results obtained with confocal, 2D, and z-STED (Fig. 3, *C* and *D*), the tubes appeared nearly full in confocal and 2D STED images, whereas z-STED significantly increased the image contrast by removing out-of-focus light originating from the top and bottom of tubes, allowing the precise visualization of such structures. Similarly, we were able to resolve and improve image resolution and contrast on toroidal structures of red blood cells (Fig. 3, *E* and *F*). In both SLBs and red blood cells, the quality of 2D STED images was deteriorated by out-of-focus contributions, which further showed the necessity of the excellent axial confinement provided by z-STED. Finally, we showed that z-STED is suitable for imaging early endocytic vesicles, another biologically important structure with a spherical topology (Fig. 3 *G*).

z-STED-FCS to investigate membrane dynamics

A unique feature of STED compared to other super-resolution microscopy techniques is the quasi-instantaneous fluorescence-switching mechanism employed for reducing the effective observation spot size and thus for increasing the spatial resolution. This speed allows the combination of this technique with spectroscopic techniques such as FCS, which requires very-high temporal resolution. A few studies applied z-STED together with FCS yet mainly focused on cytoplasmic investigations and not on membrane dynamics (45,46,52). Instead, we used here the capability of our z-STED microscope to resolve adjacent membranes (Figs. 2 and 3) to distinguish their dynamics. We first calibrated our z-STED-FCS measurements on a simple SLB system with confocal and z-STED (Fig. 4, *A* and *B*). With these measurements, we determined the effect of z-STED on diffusion time (τ_D) and apparent number of molecules in the observation area (N). We observed an approximately twofold decrease in values of the lateral transit time τ_D through the observation area for the z-STED recordings (Fig. 4 *C*), similar values of the lateral diffusion coefficient D (Fig. 4 *D*), and slightly increased normalized values of the apparent number N of fluorescent molecules in the observation area (Fig. 4 *E*). The decreased values of τ_D indicate a 30% reduction in the lateral size of the observation area for z-STED (as detailed in Materials and Methods), which is perfectly in line with the reduction observed for the bead images of Fig. 1. Knowing the lateral sizes of the confocal and the z-STED observation areas, we could calculate the diffusion coefficient of the lipid dye in SLBs (Fig. 4 *D*) and compare the average number of molecules per area unit in confocal and z-STED (Fig. 4 *E*). We

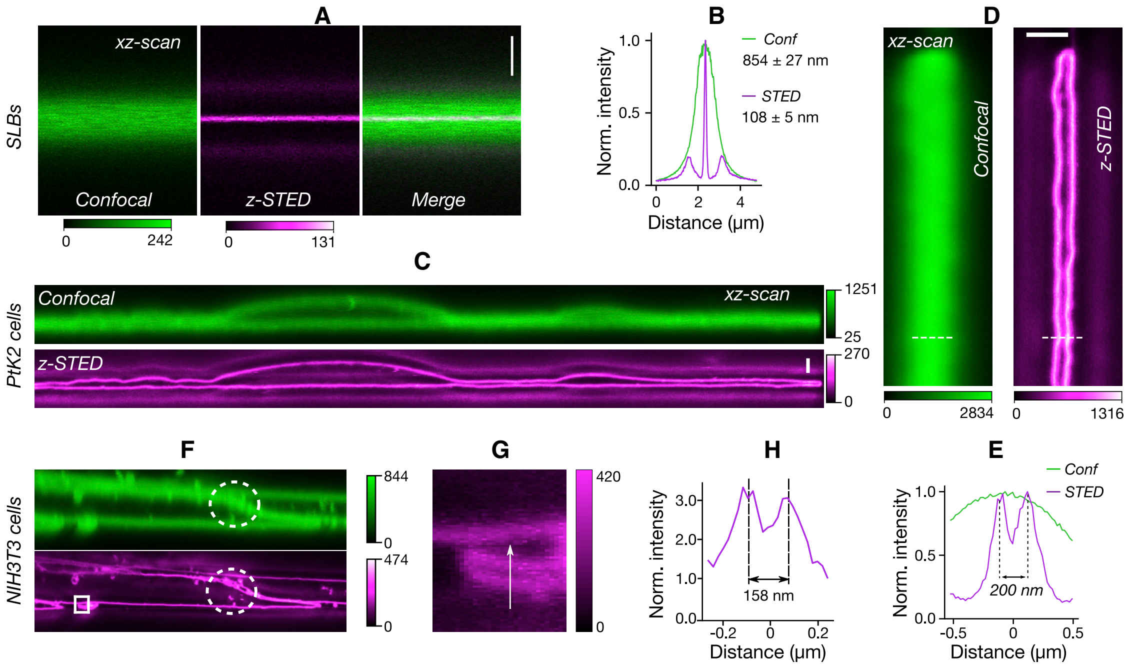

FIGURE 2 Resolving axially close-by membranes with *z*-STED. Shown are the (*A*) confocal (*left*), *z*-STED (*middle*), and merged (*right*) images of an SLB and the (*B*) confocal (*green*) and *z*-STED (*magenta*) fluorescence intensity profiles along the axial direction of the SLB shown in (*A*). (*C*) The confocal (*green*) and *z*-STED (*magenta*) images of a live PtK2 cell are given. (*D*) The top and bottom membranes of a live PtK2 cell, imaged with confocal (*left*) and *z*-STED (*right*), are shown. (*E*) Intensity profiles along the line drawn in picture (*D*) are given. (*F*) The confocal and *z*-STED images of stacked NIH-3T3 cells on top of each other are shown. *z*-STED revealed gaps between layers of membranes (*white circle*). (*G*) Zoom on the rectangular area highlighted in (*F*) is shown. (*H*) The intensity profile along the line drawn in (*G*) is given. Scale bars represent 1 μm and are oriented along the optical axis (*z* direction). To see this figure in color, go online.

found that the apparent number of molecules per area unit was slightly larger in STED than in confocal (Fig. 4 *E*), most likely because of spurious background contributions decreasing the amplitude of the STED curves (45,46,62). Using the calibration of the lateral observation area obtained with SLBs, we could compare *z*-STED and confocal FCS measurements in live cells (Fig. 4, *F–I*). Particularly, we set out to measure the diffusion speed in two close-by plasma membranes to investigate whether the molecular mobility varies between the bottom and top membranes, for instance, because of interactions with the coverslip. Confocal FCS measurements were used to measure the diffusion in the two membranes together, which could not be separated because of the limited resolution. We used *z*-STED-FCS to separately measure the diffusion of molecules in the top and bottom membranes (Fig. 4, *F* and *G*) and did not observe any change in diffusion coefficient *D* between the bottom and top membranes or between confocal and STED (Fig. 4 *H*). Moreover, there was no difference in the normalized average number of molecules between the bottom and top membranes. However, we observed that the normalized average number of molecules was two times higher in confocal than with *z*-STED (Fig. 4 *I*), which is consistent with the fact that the confocal FCS measurements covered two membranes at once, whereas only one at a time was measured with *z*-STED (see also Supporting Materials and Methods for more detailed discussions).

z-STED combined with spectral imaging to investigate membrane structure

Membrane fluidity is a crucial aspect for membrane bioactivity (63). An indirect and straightforward way to assess membrane fluidity is the use of polarity-sensitive fluorescent probes whose emission spectra shift with the polarity of the environment. Polarity in membranes generally varies with the hydration level of the bilayer, which itself is a function of lipid acyl chain packing. Compared to unsaturated lipids, saturated lipids form more tightly packed or ordered membranes where there is less space for water molecules. Recently, we have shown that the custom-synthesized environment-sensitive probe NR12S (64) is suitable for measuring membrane fluidity using 2D STED (21). We set out to test whether NR12S can also be used with *z*-STED to characterize and distinguish membrane fluidity in the close-by basal and apical membranes of adherent cells. Live PtK2 cells stained with NR12S were investigated by *z*-STED by splitting the fluorescence emission into two separate channels: a green (510–590 nm) and a red (650–730 nm) channel. Fluorescence intensity from more-ordered membrane environments increased the relative intensity

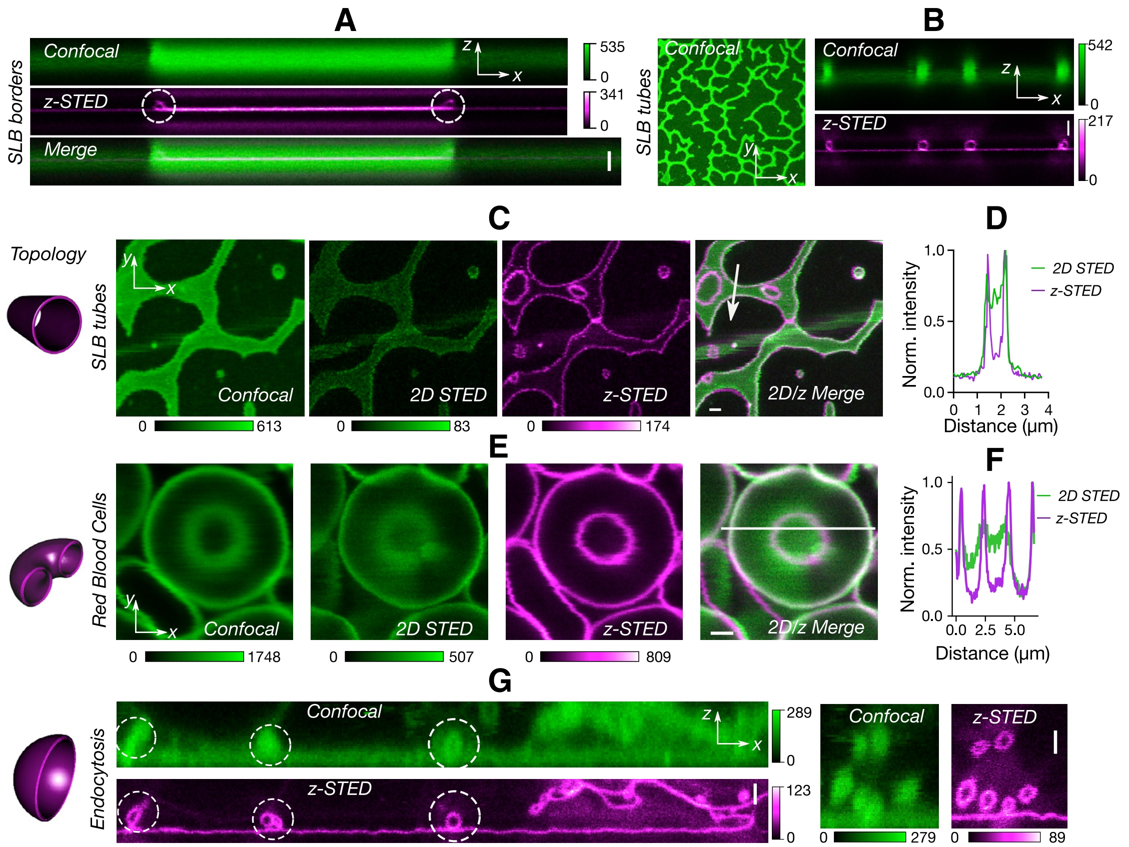

FIGURE 3 Imaging submicron membrane structures with z-STED. (*A* and *B*) The tubular structures in SLBs are the following: (*A*) confocal (*top*, *green*), z-STED (*middle*, *magenta*), and merged (*bottom*) *xz* images of a multilayer structure with tubes at the edges (*white circles*). (*B*) Lateral confocal (*left*), axial confocal (*top right*, *green*), and axial z-STED (*bottom right*, *magenta*) images of hollow networks of multilayered SLB patches are shown. (*C* and *D*) Membrane tubes in SLBs are as follows: (*C*) *xy* images in confocal, 2D, and z-STED modes. (*D*) Shown are the intensity profiles of a tube in 2D and z-STED along the line shown in (*C*). (*E* and *F*) Red blood cells with a toroidal shape are as follows: (*E*) confocal, 2D, and z-STED images (*xy* axes) and (*F*) intensity profiles of a cell in 2D and z-STED along the line shown in (*E*). (*G*) Comparison of *xz* cross sections for confocal and z-STED imaging of early endocytic vesicles (*white circles*) is given. Scale bars represent 1 μm. SLBs are labeled with Abberior STAR RED (Abberior Instruments)-phosphatidylethanolamine, and cells are labeled with Abberior STAR RED (Abberior Instruments)-cholesterol. To see this figure in color, go online.

collected in the green channel, whereas that from disordered membranes increased the relative intensity in the red channel. Using these fluorescence intensities, GP was calculated as detailed in Materials and Methods as a direct measurement of lipid ordering, with a high GP being associated with a high degree of packing. For the confocal recordings, it was impossible to discern two close-by membranes (Fig. 5, *A* and *B*); however, for z-STED recordings, we could distinguish the top and bottom membranes (Fig. 5, *A* and *B*) and measure the GP values for each separately (Fig. 5, *B* and *C*). As expected from the diffusion data (Fig. 4), we did not observe any difference between the lipid packing of the top apical and bottom basal membranes of the adherent cells. Along the line, we could also successfully distinguish the plasma membrane from endocytic vesicles and measure their GP separately (Fig. 5 *C*), which showed a lower GP for endocytic vesicles compared to the plasma membrane. Similarly, and as already highlighted in Fig. 3, z-STED also allows increasing the image contrast in *xy* images because of the reduction in out-of-focus signal, which allows the acquisition of high-contrast lateral GP images of structures such as tubes or vesicles in living cells (Fig. S5).

CONCLUSIONS

We showed here the efficient use of z-STED for image- and spectroscopy-based studies of the cellular plasma membrane. We observed fine structural details in the z-STED data that could not be resolved in the confocal counterpart. Particularly, we were able to resolve the bottom basal and top apical membranes of various cell types, which are separated by a distance well below the diffraction-limited axial resolution. Using this increased axial resolution, we imaged submicron features with different topologies, such as spheres (endocytic vesicles), tubes (in SLB patches), and tori (red blood cells). Finally, we showed that z-STED can be used together with spectroscopic tools such as FCS and spectral imaging coupled with polarity-sensitive dyes. Although the former allowed us to study the diffusion in nearby membranes, the latter allowed the observation of

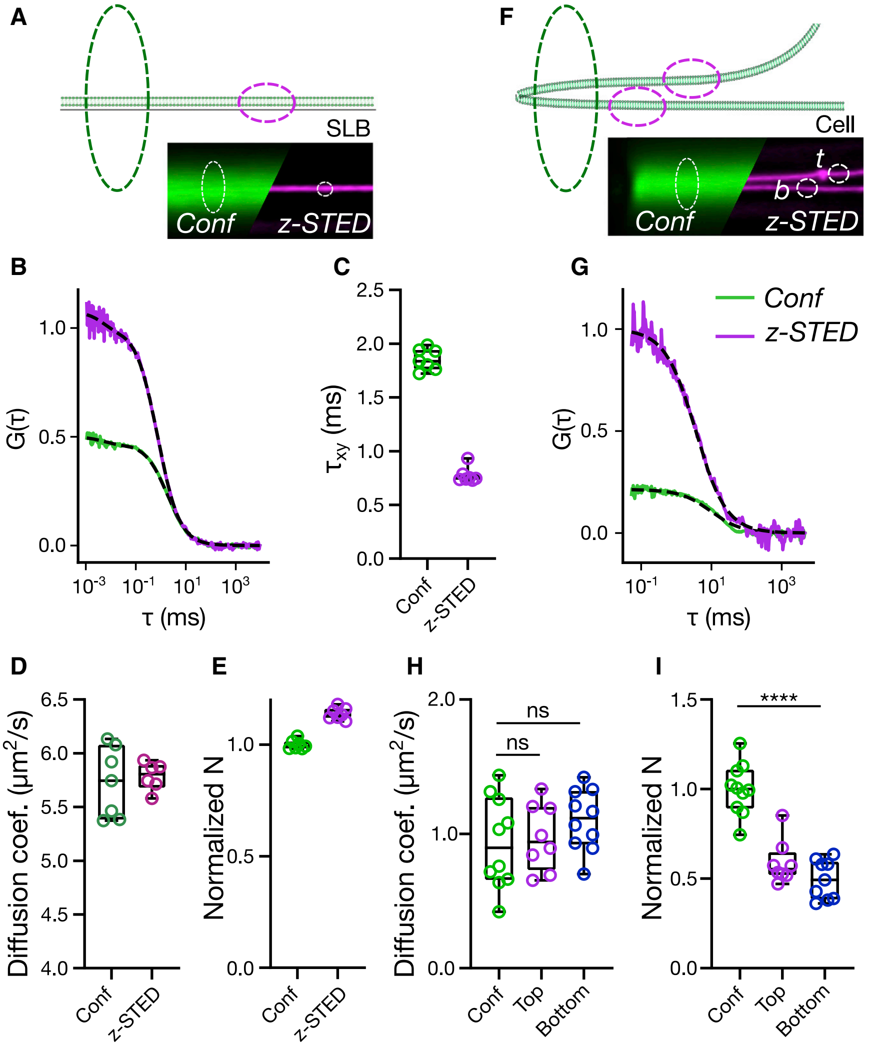

FIGURE 4 *z*-STED-FCS measurements in membranes. (*A–E*) FCS measurements in an SLB are shown. (*A*) Scheme of the experiment: confocal (*dotted green ellipsoid*) and *z*-STED-FCS (*dotted magenta ellipsoid*) measurements were performed on an SLB (lipid bilayer, *green*). Bottom right: representative confocal and *z*-STED pictures of an SLB are shown. (*B*) Representative *z*-STED and confocal FCS curves on SLBs are shown. (*C*) The average transit times measured in SLBs are given. (*D* and *E*) The measured diffusion coefficient (*D*) and average number of fluorescent molecules per surface area (*E*) normalized with confocal values measured in SLBs are given. (*F–I*) FCS measurements in living cells are shown. (*F*) Scheme of confocal and *z*-STED-FCS on two close-by membranes (*top* and *bottom*) in cells is given. (*G*) Representative *z*-STED and confocal FCS curves in cells are shown. (*H*) Diffusion coefficient and (*I*) molecular density (number of molecules normalized with observation area) in the top and bottom of the cells measured with *z*-STED-FCS or confocal FCS are shown. To see this figure in color, go online.

minute differences in lipid packing between plasma membrane and endocytic vesicles.

Membrane studies in living cells can be performed in either the bottom or top membranes. Although artifacts can occur when studying the bottom membrane because of interactions with the coverglass, measurements in the top membrane may be biased by aberrations caused by light propagation through thick sections of the cytoplasm or the nucleus. As a result, unbiased comparison between the top and bottom membranes in conventional setups is compromised. Here, we could study the top and bottom membranes of PtK2 cells, which are axially extremely close, and therefore, few to no aberrations could affect measurements in the top membrane. In this context, our results showed that the top and bottom membranes of PtK2 cells have similar biophysical properties (diffusion speed and lipid packing).

The excellent axial resolution of *z*-STED coupled with fluorescent lipid probes has a significant potential for future biological studies. We believe, for example, that the capability of our method to resolve endocytic vesicles in living cells could make it a valuable tool to study viral entry in living cells through membrane fusion or endocytosis. Furthermore, internal membranes such as endoplasmic reticulum or organelle contact sites can also be studied with this methodology.

A limitation of *z*-STED is the undepleted side lobes caused by an imperfect overlap between the excitation focus and depletion pattern, which created dim intensity shadows above and below continuous structures like membranes. These shadows were, however, created at a large distance from the focus (~800 nm) and could straightforwardly be removed using image deconvolution (as performed in Fig. S3). Such side lobes due to an undepleted signal were also an issue in other applications and could successfully be removed by adding a second STED laser pattern to the *z*-STED (20) or by engineering the depletion focus to create a better overlap between excitation and depletion, as was previously done in 4pi STED microscopes (65).

A limitation of STED microscopy is the use of high laser intensities, potentially introducing high phototoxicity and

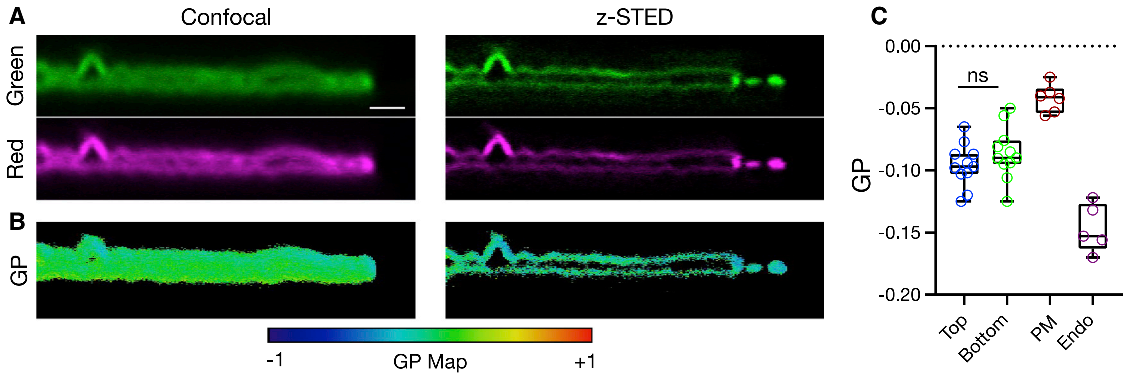

FIGURE 5 z-STED measurements of lipid order on PtK2 cells using the polarity-sensitive dye NR12S and spectral analysis. (*A*) Shown are the *xz* images of cells labeled with NR12S, confocal (*left*), and z-STED (*right*). (*B*) Shown are the *xz* confocal and z-STED GP images of the cells shown in (*A*), calculated using Eq. 6, showing two adjacent membranes clearly resolved with z-STED. (*C*) Quantification of GP for the bottom versus top membrane of the cells and whole plasma membrane versus early endocytic vesicles is shown. For quantification, the bottom or top membrane portions were selected in the GP image of the cells, and the GP values of these portions were used. For endocytic vesicles, vesicles were selected in the GP image, and the GP value of these regions was used. For the plasma membrane versus endocytic vesicles comparison, newly formed endocytic vesicles were avoided. Each data point represents a cell. Scale bars represent 1 μm. To see this figure in color, go online.

photobleaching, which usually prevent imaging of living cells over long periods of time. The photobleaching phenotype was less of an issue in our study because the photobleached fluorescent lipids were vastly replenished because of their fast diffusion. Consequently, we were able to image the same structures across multiple frames without a noticeable reduction in intensity. In this context, the use of diffusing fluorescent lipid analogs offers a robust alternative to other labeling strategies using exchangeable fluorophores (42). However, this does not rule out the existence of photobleaching.

We could perform FCS measurements in the top and bottom membranes of PtK2 cells, permitted only by the excellent (~100 nm) axial resolution provided by z-STED. However, this high precision meant that even small displacements of the membrane along the optical axis significantly biased FCS recordings, effectively reducing the maximal available acquisition times. This problem was solved in 2D using scanning FCS and an off-line correction of cellular motion (66). In the axial direction, it has, however, not yet been achieved because of the limited speed of devices generally used for axial scanning, such as piezo stages. To achieve the necessary scanning frequency (on the order of kilohertz to be faster than the measured molecular diffusion dynamics), a fast optical element like a deformable mirror could be used.

Data availability

The research materials supporting this article can be accessed by contacting the authors and will be deposited on the Oxford research archive shortly after publication.

SUPPORTING MATERIAL

Supporting Material can be found online at https://doi.org/10.1016/j.bpj.2020.04.006.

AUTHOR CONTRIBUTIONS

E.S. and A.B. designed the experiments and concepts, performed experiments, analyzed data, and generated figures. I.U. and S.G. helped with the optical setup. E.S., C.E., and M.B. helped fund the project. All authors discussed the results and wrote the manuscript.

ACKNOWLEDGMENTS

We thank the Hal Drakesmith Lab for providing red blood cells and Falk Schneider for his technical help. We thank the Wolfson Imaging Centre Oxford and the Micron Advanced Bioimaging Unit for providing the microscope facility and financial support.

We acknowledge funding from the European Research Council (AdOMIS 695140), Wolfson Foundation, the Medical Research Council (MRC; grant number MC_UU_12010 and unit programs G0902418, MC_UU_12025, and MR/K01577X/1), Engineering and Physical Sciences Research Council/MRC (grant number EP/L016052/1), the Wellcome Trust (grant 104924/14/Z/14 and strategic award 091911 Micron Advanced Bioimaging Unit), the Deutsche Forschungsgemeinschaft (research unit 1905 "Structure and function of the peroxisomal translocon"), Friedrich Schiller University Jena (Jena Excellence Cluster "Balance of the Microverse," Collaborative Research Center 1278 "Polytarget"), Jena Center of Soft Matter, Oxford University internal funds (John Fell Fund and Edward Penley Abraham Cephalosporin Fund), European Commission (MSCA IF 707348 (to I.U.)), and Wellcome Institutional Strategic Support Fund. E.S. is funded by the Newton-Katip Celebi Institutional Links grant (352333122) and SciLifeLab fellowship.

REFERENCES

1. Cho, W., and R. V. Stahelin. 2005. Membrane-protein interactions in cell signaling and membrane trafficking. *Annu. Rev. Biophys. Biomol. Struct.* 34:119–151.

2. Jacobson, K., P. Liu, and B. C. Lagerholm. 2019. The lateral organization and mobility of plasma membrane components. *Cell.* 177:806–819.

3. Baumgart, F., A. M. Arnold, ..., G. J. Schütz. 2016. Varying label density allows artifact-free analysis of membrane-protein nanoclusters. *Nat. Methods.* 13:661–664.

4. Sezgin, E., I. Levental, …, C. Eggeling. 2017. The mystery of membrane organization: composition, regulation and roles of lipid rafts. *Nat. Rev. Mol. Cell Biol.* 18:361–374.

5. Sezgin, E. 2017. Super-resolution optical microscopy for studying membrane structure and dynamics. *J. Phys. Condens. Matter.* 29:273001.

6. Stone, M. B., S. A. Shelby, and S. L. Veatch. 2017. Super-resolution microscopy: shedding light on the cellular plasma membrane. *Chem. Rev.* 117:7457–7477.

7. Stone, M. B., S. A. Shelby, …, S. L. Veatch. 2017. Protein sorting by lipid phase-like domains supports emergent signaling function in B lymphocyte plasma membranes. *eLife.* 6::e19891.

8. Sengupta, P., T. Jovanovic-Talisman, …, J. Lippincott-Schwartz. 2011. Probing protein heterogeneity in the plasma membrane using PALM and pair correlation analysis. *Nat. Methods.* 8:969–975.

9. Shelby, S. A., D. Holowka, …, S. L. Veatch. 2013. Distinct stages of stimulated FcεRI receptor clustering and immobilization are identified through superresolution imaging. *Biophys. J.* 105:2343–2354.

10. Owen, D. M., C. Rentero, …, K. Gaus. 2010. PALM imaging and cluster analysis of protein heterogeneity at the cell surface. *J. Biophotonics.* 3:446–454.

11. Owen, D. M., D. J. Williamson, …, K. Gaus. 2012. Sub-resolution lipid domains exist in the plasma membrane and regulate protein diffusion and distribution. *Nat. Commun.* 3:1256.

12. Huang, B., W. Wang, …, X. Zhuang. 2008. Three-dimensional super-resolution imaging by stochastic optical reconstruction microscopy. *Science.* 319:810–813.

13. Shtengel, G., J. A. Galbraith, …, H. F. Hess. 2009. Interferometric fluorescent super-resolution microscopy resolves 3D cellular ultrastructure. *Proc. Natl. Acad. Sci. USA.* 106:3125–3130.

14. Bon, P., J. Linarès-Loyez, …, L. Cognet. 2018. Self-interference 3D super-resolution microscopy for deep tissue investigations. *Nat. Methods.* 15:449–454.

15. Gwosch, K. C., J. K. Pape, …, S. W. Hell. 2020. MINFLUX nanoscopy delivers 3D multicolor nanometer resolution in cells. *Nat. Methods.* 17:217–224.

16. Chizhik, A. I., J. Rother, …, J. Enderlein. 2014. Metal-induced energy transfer for live cell nanoscopy. *Nat. Photonics.* 8:124–127.

17. Karedla, N., A. I. Chizhik, …, J. Enderlein. 2014. Single-molecule metal-induced energy transfer (smMIET): resolving nanometer distances at the single-molecule level. *ChemPhysChem.* 15:705–711.

18. Ghosh, A., A. Sharma, …, J. Enderlein. 2019. Graphene-based metal-induced energy transfer for sub-nanometre optical localization. *Nat. Photonics.* 13:860–865.

19. Chizhik, A. M., D. Ruhlandt, …, J. Enderlein. 2017. Three-dimensional reconstruction of nuclear envelope architecture using dual-color metal-induced energy transfer imaging. *ACS Nano.* 11:11839–11846.

20. Hein, B., K. I. Willig, and S. W. Hell. 2008. Stimulated emission depletion (STED) nanoscopy of a fluorescent protein-labeled organelle inside a living cell. *Proc. Natl. Acad. Sci. USA.* 105:14271–14276.

21. Sezgin, E., F. Schneider, …, C. Eggeling. 2017. Polarity-sensitive probes for superresolution stimulated emission depletion microscopy. *Biophys. J.* 113:1321–1330.

22. Schneider, F., D. Waithe, …, E. Sezgin. 2017. Diffusion of lipids and GPI-anchored proteins in actin-free plasma membrane vesicles measured by STED-FCS. *Mol. Biol. Cell.* 28:1507–1518.

23. Eggeling, C., C. Ringemann, …, S. W. Hell. 2009. Direct observation of the nanoscale dynamics of membrane lipids in a living cell. *Nature.* 457:1159–1162.

24. Sezgin, E., F. Schneider, …, C. Eggeling. 2019. Measuring nanoscale diffusion dynamics in cellular membranes with super-resolution STED-FCS. *Nat. Protoc.* 14:1054–1083.

25. Fahey, P. F., D. E. Koppel, …, W. W. Webb. 1977. Lateral diffusion in planar lipid bilayers. *Science.* 195:305–306.

26. Schwille, P., J. Korlach, and W. W. Webb. 1999. Fluorescence correlation spectroscopy with single-molecule sensitivity on cell and model membranes. *Cytometry.* 36:176–182.

27. Treanor, B., D. Depoil, …, F. D. Batista. 2010. The membrane skeleton controls diffusion dynamics and signaling through the B cell receptor. *Immunity.* 32:187–199.

28. Mii, Y., and M. Taira. 2009. Secreted frizzled-related proteins enhance the diffusion of Wnt ligands and expand their signalling range. *Development.* 136:4083–4088.

29. Takada, R., Y. Mii, …, S. Takada. 2018. Assembly of protein complexes restricts diffusion of Wnt3a proteins. *Commun. Biol.* 1:165.

30. Bagawath-Singh, S., E. Staaf, …, S. Johansson. 2016. Cytokines induce faster membrane diffusion of MHC class I and the Ly49A receptor in a subpopulation of natural killer cells. *Front. Immunol.* 7:16.

31. Chojnacki, J., D. Waithe, …, C. Eggeling. 2017. Envelope glycoprotein mobility on HIV-1 particles depends on the virus maturation state. *Nat. Commun.* 8:545.

32. Schneider, F., D. Waithe, …, C. Eggeling. 2018. Nanoscale spatiotemporal diffusion modes measured by simultaneous confocal and stimulated emission depletion nanoscopy imaging. *Nano Lett.* 18:4233–4240.

33. Vicidomini, G., H. Ta, …, C. Eggeling. 2015. STED-FLCS: an advanced tool to reveal spatiotemporal heterogeneity of molecular membrane dynamics. *Nano Lett.* 15:5912–5918.

34. Benda, A., Y. Ma, and K. Gaus. 2015. Self-calibrated line-scan STED-FCS to quantify lipid dynamics in model and cell membranes. *Biophys. J.* 108:596–609.

35. Hedde, P. N., R. M. Dörlich, …, G. U. Nienhaus. 2013. Stimulated emission depletion-based raster image correlation spectroscopy reveals biomolecular dynamics in live cells. *Nat. Commun.* 4:2093.

36. Bianchini, P., F. Cardarelli, …, R. Bizzarri. 2014. Nanoscale protein diffusion by STED-based pair correlation analysis. *PLoS One.* 9:e99619.

37. Sarangi, N. K., I. I. P, …, J. K. Basu. 2016. Super-resolution stimulated emission depletion-fluorescence correlation spectroscopy reveals nanoscale membrane reorganization induced by pore-forming proteins. *Langmuir.* 32:9649–9657.

38. Klar, T. A., S. Jakobs, …, S. W. Hell. 2000. Fluorescence microscopy with diffraction resolution barrier broken by stimulated emission. *Proc. Natl. Acad. Sci. USA.* 97:8206–8210.

39. Osseforth, C., J. R. Moffitt, …, J. Michaelis. 2014. Simultaneous dual-color 3D STED microscopy. *Opt. Express.* 22:7028–7039.

40. Tønnesen, J., V. V. G. K. Inavalli, and U. V. Nägerl. 2018. Super-resolution imaging of the extracellular space in living brain tissue. *Cell.* 172:1108–1121.e15.

41. Zdankowski, P., D. McGloin, and J. R. Swedlow. 2019. Full volume super-resolution imaging of thick mitotic spindle using 3D AO STED microscope. *Biomed. Opt. Express.* 10:1999–2009.

42. Spahn, C., J. B. Grimm, …, M. Heilemann. 2019. Whole-cell, 3D, and multicolor STED imaging with exchangeable fluorophores. *Nano Lett.* 19:500–505.

43. Heine, J., C. A. Wurm, …, G. Donnert. 2018. Three dimensional live-cell STED microscopy at increased depth using a water immersion objective. *Rev. Sci. Instrum.* 89:053701.

44. Harke, B., C. K. Ullal, …, S. W. Hell. 2008. Three-dimensional nanoscopy of colloidal crystals. *Nano Lett.* 8:1309–1313.

45. Kastrup, L., H. Blom, …, S. W. Hell. 2005. Fluorescence fluctuation spectroscopy in subdiffraction focal volumes. *Phys. Rev. Lett.* 94:178104.

46. Ringemann, C., B. Harke, …, C. Eggeling. 2009. Exploring single-molecule dynamics with fluorescence nanoscopy. *New J. Phys.* 11:103054.

47. Gao, P., and G. U. Nienhaus. 2017. Precise background subtraction in stimulated emission double depletion nanoscopy. *Opt. Lett.* 42:831–834.

48. Deng, S., L. Liu, …, Z. Xu. 2009. Investigation of the influence of the aberration induced by a plane interface on STED microscopy. *Opt. Express.* 17:1714–1725.
49. Deng, S., L. Liu, …, Z. Xu. 2010. Effects of primary aberrations on the fluorescence depletion patterns of STED microscopy. *Opt. Express.* 18:1657–1666.
50. Gould, T. J., D. Burke, …, M. J. Booth. 2012. Adaptive optics enables 3D STED microscopy in aberrating specimens. *Opt. Express.* 20:20998–21009.
51. Lenz, M. O., H. G. Sinclair, …, P. M. French. 2014. 3-D stimulated emission depletion microscopy with programmable aberration correction. *J. Biophotonics.* 7:29–36.
52. Barbotin, A., S. Galiani, …, M. J. Booth. 2019. Adaptive optics allows STED-FCS measurements in the cytoplasm of living cells. *Opt. Express.* 27:23378–23395.
53. Barbotin, A. 2020. Alignment of an SLM-based STED depletion beam. *Zenodo* https://doi.org/10.5281/zenodo.3567748.
54. Clausen, M. P., E. Sezgin, …, C. Eggeling. 2015. A straightforward approach for gated STED-FCS to investigate lipid membrane dynamics. *Methods.* 88:67–75.
55. Galiani, S., D. Waithe, …, C. Eggeling. 2016. Super-resolution microscopy reveals compartmentalization of peroxisomal membrane proteins. *J. Biol. Chem.* 291:16948–16962.
56. Patton, B. R., D. Burke, …, M. J. Booth. 2015. Is phase-mask alignment aberrating your STED microscope? *Methods Appl. Fluoresc.* 3:024002.
57. Sezgin, E., and P. Schwille. 2011. Fluorescence techniques to study lipid dynamics. *Cold Spring Harb. Perspect. Biol.* 3:a009803.
58. Darwich, Z., A. S. Klymchenko, …, Y. Mély. 2012. Detection of apoptosis through the lipid order of the outer plasma membrane leaflet. *Biochim. Biophys. Acta.* 1818:3048–3054.
59. Sezgin, E., D. Waithe, …, C. Eggeling. 2015. Spectral imaging to measure heterogeneity in membrane lipid packing. *ChemPhysChem.* 16:1387–1394.
60. Antonello, J., E. B. Kromann, …, M. J. Booth. 2016. Coma aberrations in combined two- and three-dimensional STED nanoscopy. *Opt. Lett.* 41:3631–3634.
61. Nawaz, M., and F. Fatima. 2017. Extracellular vesicles, tunneling nanotubes, and cellular interplay: synergies and missing links. *Front. Mol. Biosci.* 4:50.
62. Hess, S. T., and W. W. Webb. 2002. Focal volume optics and experimental artifacts in confocal fluorescence correlation spectroscopy. *Biophys. J.* 83:2300–2317.
63. Veya, L., J. Piguet, and H. Vogel. 2015. Single molecule imaging deciphers the relation between mobility and signaling of a prototypical G protein-coupled receptor in living cells. *J. Biol. Chem.* 290:27723–27735.
64. Kucherak, O. A., S. Oncul, …, A. S. Klymchenko. 2010. Switchable nile red-based probe for cholesterol and lipid order at the outer leaflet of biomembranes. *J. Am. Chem. Soc.* 132:4907–4916.
65. Hao, X., E. S. Allgeyer, …, J. Bewersdorf. 2015. Point-spread function optimization in isoSTED nanoscopy. *Opt. Lett.* 40:3627–3630.
66. Ries, J., S. R. Yu, …, P. Schwille. 2009. Modular scanning FCS quantifies receptor-ligand interactions in living multicellular organisms. *Nat. Methods.* 6:643–645.

Biophysical Journal, Volume *118*

Supplemental Information

z-STED Imaging and Spectroscopy to Investigate Nanoscale Membrane Structure and Dynamics

Aurélien Barbotin, Iztok Urbančič, Silvia Galiani, Christian Eggeling, Martin Booth, and Erdinc Sezgin

Supplementary Information

Membrane motion observed with imaging and FCS

In cells, membrane motion limited the maximum observation time in FCS measurements. Indeed, the membranes of living cells move with time. Because of the extremely small axial size of the Gaussian observation volume (approximately 100 nm FWHM), even small displacements of the membrane induce significant artefacts in FCS curves. To evaluate this, we recorded a series of 6 movies of the top and bottom membranes of Ptk2 cells (50 frames acquired with a framerate of 3.2 s, at a STED power of 50 mW). In each movie, we manually selected three points in each membrane, the position of which was measured by Gaussian fitting (Figure S1, A-C). Plotting the mean displacement with time (Figure S1D) and comparing it with the displacement measured in a SLB revealed that cellular membranes move significantly more than SLBs. We quantified this displacement with linear fits, the slope of which we used as an estimate of membrane motion (0.7 and 0.6 nm/s median shift for top and bottom respectively, while only 0.01 nm/s for SLBs). Non-zero intercepts were caused by the limited localisation precision induced by noise. The changes in intensity resulting from varying membrane position within the focus precluded long z-STED-FCS measurements in cells (Figure S1E), while no such effect was visible in SLBs (Figure S1F).

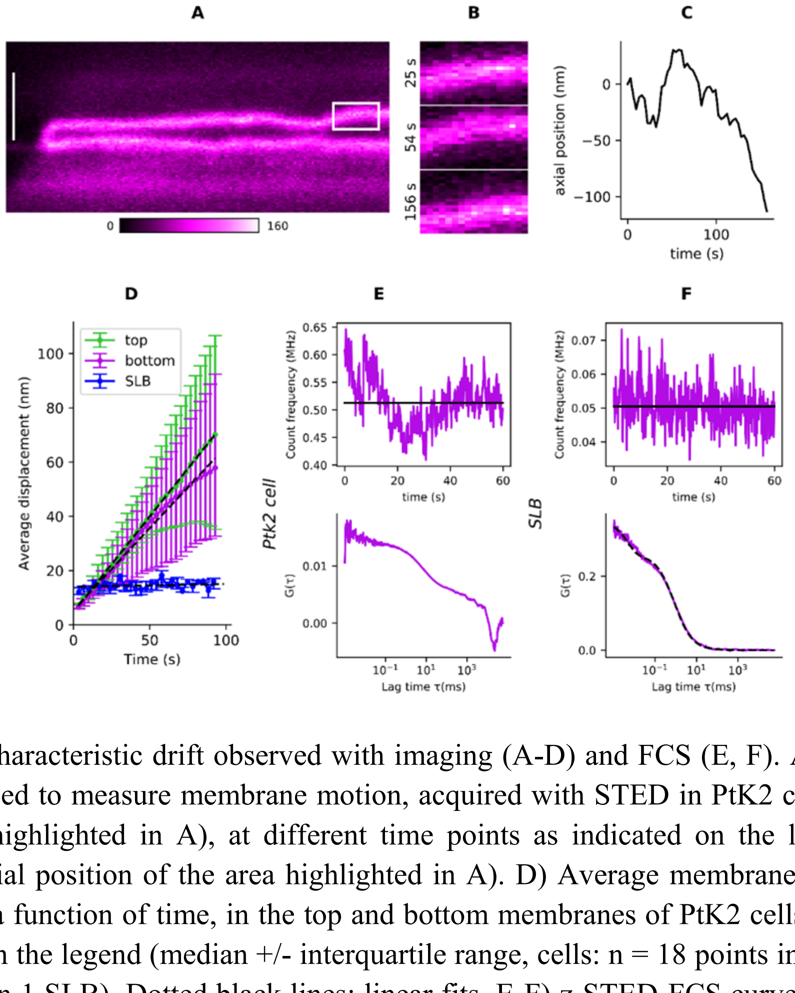

Figure S1. Characteristic drift observed with imaging (A-D) and FCS (E, F). A) First frame of a movie used to measure membrane motion, acquired with STED in PtK2 cells. B) Zoom on the area highlighted in A), at different time points as indicated on the left. C) Time-dependent axial position of the area highlighted in A). D) Average membrane displacement measured as a function of time, in the top and bottom membranes of PtK2 cells and in SLBs as indicated in the legend (median +/- interquartile range, cells: n = 18 points in 6 cells, SLB: n = 3 points in 1 SLB). Dotted black lines: linear fits. E-F) z-STED-FCS curves acquired for 60 s in E) the top membrane of a Ptk2 cell and F) in an SLB.

Representative FCS curves

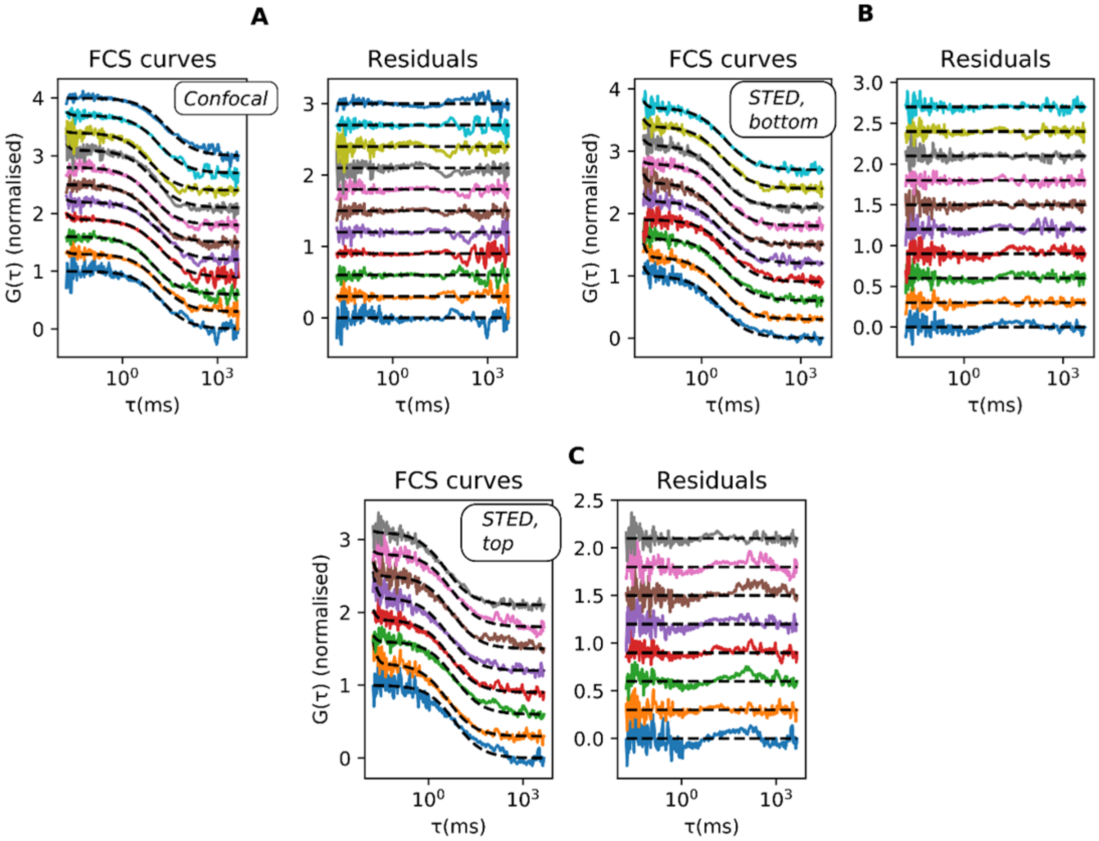

Figure S2. FCS curves (left) and corresponding residuals (right) acquired in cells, in A) confocal mode, B) STED in the bottom membrane and C) STED in the top membrane.

Image deconvolution

Shallow side lobes due to undepleted fluorescence signal were particularly visible when imaging membranes with z-STED. A solution to remove them is to use image deconvolution. First, the point spread function (PSF) of the microscope was estimated. From images of SLBs, we estimated the axial intensity profile of the z-STED PSF (see Figure 2C in main text). The lateral intensity profile was assumed to be Gaussian, with a lateral size estimated from FCS recordings in SLBs estimated to be equal to 160 nm (see Methods section). The PSF was then reconstructed as follows:

$$PSF(x, z) = I(z)\exp(-4\log(2)x^2/\omega^2) \tag{1}$$

Where *I(z)* is the axial intensity profile and *ω* the lateral FWHM of the PSF. Using this PSF (Figure S1A), images were deconvolved employing the Richardson-Lucy algorithm (20 iterations) in Python using the library scikit-image[1]. Deconvolution improved the sharpness of the images and efficiently suppressed the side lobe contributions (Figure S3).

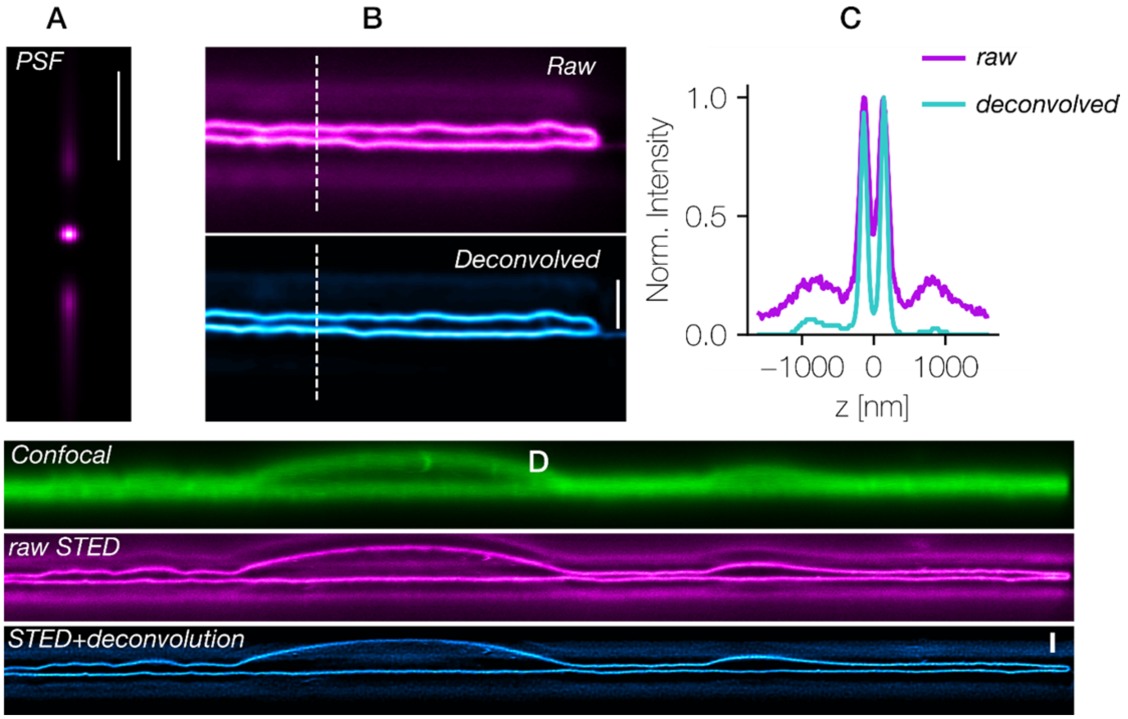

Figure S3. Suppressing contribution from side lobes due to undepleted fluorescence signal using image deconvolution. A) PSF of the z-STED microscope, estimated from imaging and FCS of SLBs. B, C) Raw (magenta, top B) and deconvolved (blue, bottom B) of z-STED images of two axially close-by membranes along with intensity profiles (C) along the line marked in B. D) Deconvolution of a z-STED image of a whole Ptk2 cell: comparison of confocal (top, green), raw STED (middle, magenta) and deconvolved z-STED (bottom, cyan). Scale bars 1 µm.

z-STED imaging of fluorescent lipids in different cell types

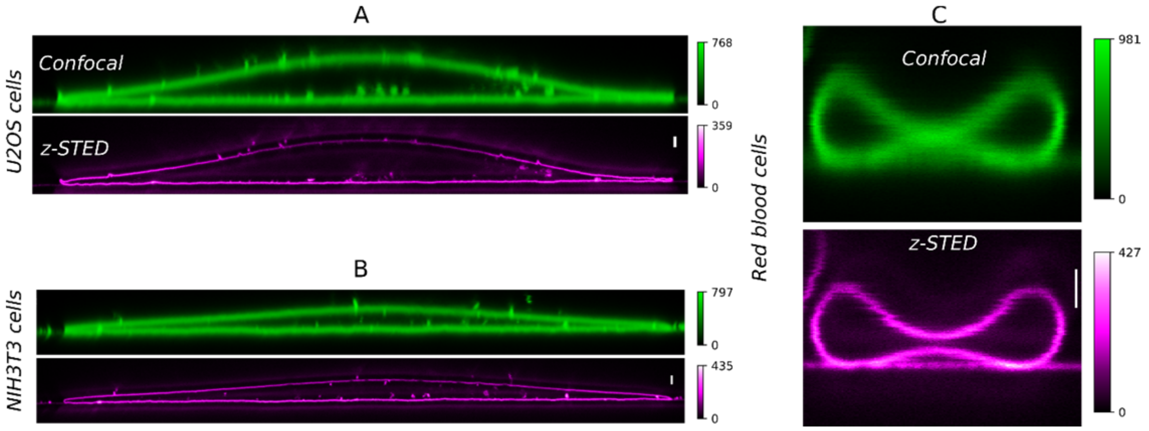

Figure S4. Imaging different cell types with z-STED. *xz* images of live A) U2OS, B) NIH3T3, and C) red blood cells, labelled with Abberior Star Red-PEG-Cholesterol. Scale bars 1 µm.

***xy* improvement of GP imaging**

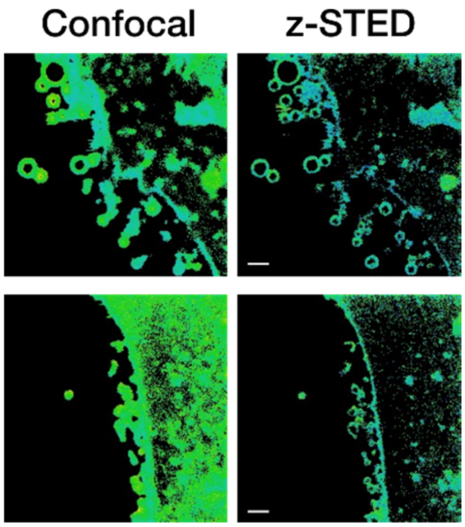

Figure S5. *xy* confocal and STED GP images of cells labelled with NR12S probe, showing how increase in contrast in axial direction helps visualizing and characterizing the small vesicles that cannot be resolved with confocal. Scale bars are 1 μm.

Theoretical analysis of FCS experiments in two close-by membranes

We compared the results obtained when doing FCS in the top and bottom membranes with z-STED as well as on both at the same time with confocal FCS. It is possible to analytically predict the outcomes of such measurements, if they satisfy three assumptions:

1. The intensity fluctuations in the top and bottom membranes are statistically independent.
2. The top and bottom membranes are close enough so that the confocal cross section is identical at both positions.
3. The label concentration and diffusion speed of the top and bottom membranes are strictly identical.

Following these assumptions, the time-dependent intensity recorded in confocal mode by the detector can be written as:

$$I(t) = I_t(t) + I_b(t) \tag{2}$$

Where *I(t)* is the time-dependent intensity recorded by the detector, $I_t(t)$ is the intensity emitted at the top membrane and $I_b(t)$ the intensity emitted at the bottom membrane.

The autocorrelation function of the time trace fluctuations writes as:

$$G(\tau) = \frac{\langle \delta I(t) \delta I(t+\tau) \rangle}{\langle I(t) \rangle^2} \tag{3}$$

where $\langle . \rangle$ denotes the time-averaging operator, and $\delta I(t) = I(t) - \langle I(t) \rangle$ represents the temporal intensity fluctuations. Replacing equation 2 in equation 3 and expanding yields:

$$G(\tau) = \frac{\langle \delta I_t(t) \delta I_t(t+\tau) \rangle + \langle \delta I_b(t) \delta I_b(t+\tau) \rangle + \langle \delta I_t(t) \delta I_b(t+\tau) \rangle + \langle \delta I_b(t) \delta I_t(t+\tau) \rangle}{\langle I_t(t) \rangle^2 + \langle I_b(t) \rangle^2 + 2\langle I_b(t) \rangle \langle I_t(t) \rangle} \tag{4}$$

This expression can be greatly simplified using our above assumption. Independence of the fluctuations in the top and bottom membranes (assumption 1) involves that $\langle \delta I_t(t) \delta I_b(t+\tau) \rangle = \langle \delta I_b(t) \delta I_t(t+\tau) \rangle = 0$. Assumptions 2 and 3 state that the average intensities and intensities fluctuations are identical in top and bottom membranes and we can conclude that $\langle I_t(t)^2 \rangle = \langle I_b(t)^2 \rangle$ and $\langle \delta I_t(t) \delta I_t(t+\tau) \rangle = \langle \delta I_b(t) \delta I_b(t+\tau) \rangle$. As such, equation 4 can be simplified as:

$$G(\tau) = \frac{2\langle \delta I_t(t) \delta I_t(t+\tau) \rangle}{4\langle I_t(t) \rangle^2} = \frac{1}{2} G_t(\tau) \tag{5}$$

Where $G_t(\tau)$ is the autocorrelation function obtained if only the top (or bottom) membrane was present. Equation (5) thus means that FCS measurements performed on two axially close-by membranes leaves the transit times (and consequently apparent diffusion coefficient) unchanged, but divides the amplitude by a factor two. This means that the apparent number of fluorescent molecules is twice higher when measuring two membranes at once than when

measuring membranes separately. This matches our experimental results (Figure 4, H and I in main text).

References

1. Stéfan van der Walt, Johannes L. Schönberger, Juan Nunez-Iglesias, François Boulogne, Joshua D. Warner, Neil Yager, Emmanuelle Gouillart, Tony Yu and the scikit-image contributors. scikit-image: Image processing in Python. PeerJ 2:e453 (2014)
